# Relational Reasoning Across Cultures: Consistency and Variation in South Korea, the United States, and China

**DOI:** 10.3390/bs16081406

**Published:** 2026-08-17

**Authors:** Alexandra Carstensen, Minju Kim, Youngon Choi, Caren M. Walker

**Affiliations:** 1 Psychology, School of Social and Behavioral Sciences, Arizona State University, Glendale, AZ 85306, USA; 2 Department of Psychology, Chapman University, Orange, CA 92866, USA; minjukim@chapman.edu; 3 Department of Psychology, Chung-Ang University, Seoul 06974, Republic of Korea; yochoi@cau.ac.kr; 4 Department of Psychology, UC San Diego, La Jolla, CA 92093, USA; cmwalker@ucsd.edu

**Keywords:** cognitive development, causal learning, relational reasoning, language, culture, context, South Korea

## Abstract

Abstract reasoning in early childhood is often described as following a “relational shift,” over which children become increasingly sensitive to relations. However, recent work has challenged the generality of this account, showing that children in the US and China follow distinct trajectories in a relational match-to-sample task. This difference aligns with multiple cultural and linguistic factors implicated in relational reasoning, where the US and China appear at opposite ends of a continuum, focusing on objects (US) versus relations (CN). This raises the possibility that differences in culture and language may shape variation in the development of abstract reasoning. Here, we examine the early development of relational reasoning in South Korea, a cultural and linguistic context that lies between the US and China. If environmental factors shape relational reasoning, we may therefore observe a distinct developmental trajectory in Korean children. We find that Korean 18–30-month-olds successfully infer the *same* relation, suggesting early consistency across the three contexts. Beginning at 3 years, however, Korean children show no evidence of cognitive bias, making them distinct from peers in both the US (object bias) and China (relational bias), but perform more similarly to US children, suggesting nuanced variation in abstract reasoning development across contexts.

## 1. Introduction

How do people come to reason abstractly, conceiving of objects not just in terms of properties, like color and shape, but in terms of more general similarity, like being the *same* color or shape? Abstract reasoning in early childhood is often described as following a “relational shift,” over which children become less focused on object properties and surface features, and increasingly sensitive to relations (e.g., Gentner, 1988; Gentner & Rattermann, 1991; Gentner et al., 1995). Yet recent cross-cultural work challenges the universality of this shift, suggesting that the developmental trajectory of relational reasoning critically depends on input in children’s learning environments (e.g., Carstensen et al., 2019).

**Figure 1:**
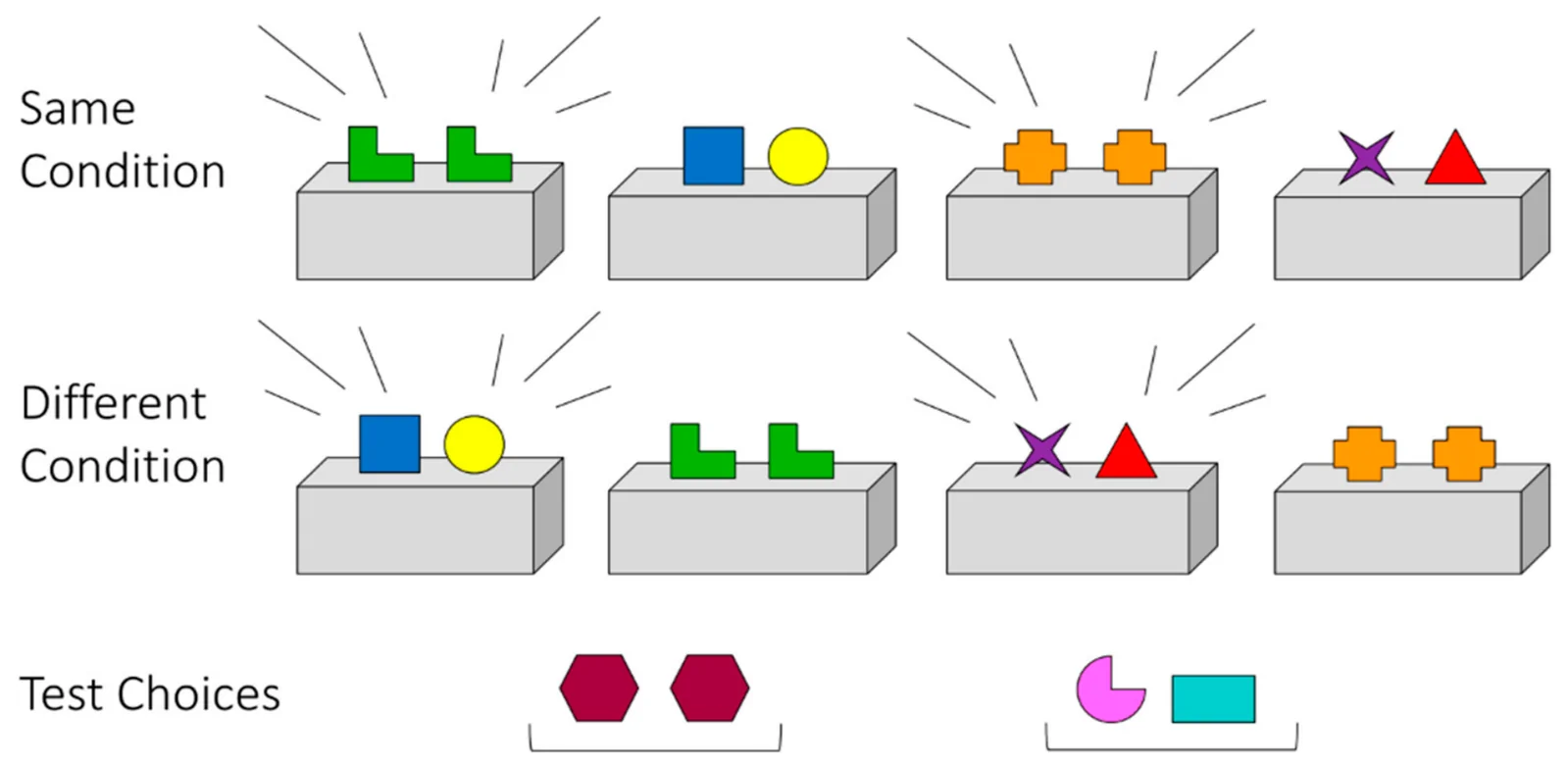
Schematic of the causal Relational Match-to-Sample training (top two rows) and test trial (bottom), reproduced from Carstensen et al. (2019). In the same condition, music played when the pairs of blocks were identical, so the correct relational choice is the identical block pair shown here on the left. The different block pair on the right was the correct choice under the different condition (though the specific blocks and their left/right placement varied across participants). Note that the test choice blocks never appeared during the training, requiring the child to abstract the relations between the blocks to select the relational answer.

In the current study, we focus on a causal relational match-to-sample (cRMTS) paradigm that tracks children’s ability to infer and generalize relational structure (Walker & Gopnik, 2014). In this task, children see four pairs of blocks placed on a toy that plays music for only two of the four pairs. In the *same* condition, the two causal pairs match in color and shape; in the *different* condition, the two causal pairs mismatch in both features (Figure 1). On a subsequent test, children choose between the same and different novel pairs to activate the toy. To succeed, children must therefore ignore salient surface features and select the novel pair that is relationally consistent with training.

Interestingly, performance trajectories in the US and China diverge on this task. While children in the US show a ‘U-shaped’ pattern between 18 and 48 months—early success, followed by a dip in performance, and later recovery—children in China show consistently strong performance across the same window (Carstensen et al., 2019). According to the authors, children across cultural contexts have genuine relational concepts from a very young age, but learned biases can lead them to privilege or neglect relational hypotheses as potential solutions. Specifically, relational and object-based representations are both available early in development, but the prior expectations supporting one or the other possibility are shaped by children’s accumulated experience (i.e., rational learner account; Walker et al., 2016). While toddlers begin with relatively flat priors and readily infer relational causes, older children (in the US) increasingly privilege hypotheses consistent with their expanding focus on individual objects and their properties. Thus, cross-cultural variability in early input may alter these developing priors, leading to differences in how readily children infer a relational versus a feature-based solution.

Importantly, the rational learner account is intended as a computational-level explanation of how children integrate experience to guide inference, rather than a mechanistic account of cognitive development. It emphasizes that input matters for inference—but also highlights important open questions about *which* aspects of children’s environments shift their priors in different directions and produce these distinct trajectories. These are complex questions for both theoretical and empirical reasons. Theoretically, the relevant factor or factors are difficult to distinguish because relational reasoning is composed of several developing capacities, including memory retrieval, structural abstraction, and representation of relational information, all of which unfold in a context where attention, working memory, and response monitoring and evaluation play supporting roles. Empirically, evidence on variation in relational reasoning across cultural and linguistic contexts remains largely correlational, and observations of relational reasoning are often collected without accompanying measures of these related factors, leaving a limited empirical basis for preferring one candidate factor, or type of factor, over another. Nevertheless, researchers have identified multiple factors that could explain these observed cross-cultural differences in relational reasoning, including those rooted in language learning (Carstensen et al., 2019; Hoyos et al., 2016), visual attention (Christie et al., 2020; Kuwabara & Smith, 2012, 2016), social cognition (Duffy et al., 2009), and cognitive development (e.g., Richland et al., 2010).

First, English-learning children exhibit rapid noun learning, potentially biasing attention temporarily toward object kinds and properties (Fernald & Morikawa, 1993; Gopnik et al., 1996; Hoyos et al., 2016). In contrast, Mandarin Chinese learners show more verb-rich, action-oriented word learning, which may support attention to interactions and roles, sustaining relational responding as a result (Chan et al., 2011; Frank et al., 2021; Tardif et al., 1997; Yuan et al., 2024). On this account, differences in early lexical experience could help explain why US learners show a decline in relational responding while Chinese learners do not (Carstensen et al., 2019; Hoyos et al., 2016).

Another set of factors concerns cultural differences in attentional and interpretive biases (e.g., Cao et al., 2024; Christie et al., 2020; Kuwabara & Smith, 2012, 2016). East Asian adults and children show stronger tendencies to prioritize contextual information and relations (a holistic bias) in visual scenes compared to people from Western cultures, who are more likely to attend to focal objects (an analytic bias; Boduroglu et al., 2009; Jurkat et al., 2024; Masuda et al., 2008; Masuda, 2017; Masuda & Nisbett, 2001). These tendencies emerge early (Kuwabara & Smith, 2012, 2016; Imada et al., 2013) and appear to be culturally transmitted by caregivers to their children (Senzaki et al., 2016; Senzaki & Shimizu, 2022). For example, when Canadian and Japanese mothers described visual scenes to their children, Japanese mothers focused more on contextual information, while Canadian mothers tended to emphasize focal objects. Although Japanese and Canadian 7–9-year-olds did not show distinct attentional biases when assessed individually, cross-cultural differences in attention emerged when the children interacted with their parents (Senzaki et al., 2016). Similarly, in social reasoning, Chinese school-aged children are more likely to attend to situational information when interpreting others’ behavior, compared to US children, who more often reference dispositions or intent (Seiver, 2013; see Ge (2026) for a recent review). This cross-cultural variability in sensitivity to context may generalize to shape reasoning across tasks.

Finally, domain-general cognitive skills, like executive function, have been linked to success in relational reasoning—specifically, the ability to suppress attention to salient, but irrelevant features (Richland et al., 2010). Indeed, relational reasoning has often been viewed as a core component of general intelligence and broader cognitive ability, given its involvement in searching for, aligning, and abstracting structural relations across contexts (Demetriou et al., 2025, 2026; Kazali et al., 2024).

Cross-cultural differences in these control processes have been documented (e.g., stronger executive function among Chinese preschoolers than in their Canadian peers, Sabbagh et al., 2006; see also Lahat et al., 2010; Schirmbeck et al., 2020), and may themselves be scaffolded by cultural factors (Imada et al., 2013). Together, these findings suggest that the US and China sit at opposite ends of a continuum, with greater object focus in the US and greater relation focus in China.

Here, we measure early relational reasoning in a third context that represents a cultural and linguistic middle ground: Korean-learning children growing up in South Korea. Although small-sample naturalistic studies report earlier verb than noun learning, or comparable rates for verbs and nouns (S. Choi & Gopnik, 1995; M. Kim et al., 2000), recent large-scale standardized vocabulary data from over 1300 Korean-learning children show that by around 30 months, their vocabularies are noun-biased (Frank et al., 2021). On the other hand, prior work has also shown that, like Chinese (Hung & Chang, 2023), early Korean caregiver speech is often action-oriented and relatively verb-friendly (S. Choi & Gopnik, 1995; M. Kim et al., 2000; M. Kim et al., 2026; Tardif et al., 1997, 1999), which contrasts with American English input that is naming-oriented and noun-friendly (Fernald & Morikawa, 1993; Gopnik et al., 1996; M. Kim et al., 2000; Poulin-Dubois et al., 1995). Thus, measured lexical composition and input point in different directions, providing an ideal setting for testing language-based accounts. Specifically, if the verb-dominant input from Korean caregivers is sufficient to facilitate the processing of relational information, Korean learners may exhibit a similar trajectory to that of Chinese children, with no observable decline in relational reasoning. By contrast, if the acquisition of nouns itself is a key factor (Hoyos et al., 2016), driving US 3-year-olds’ attention away from relational properties, Korean children aged 30 months and older may exhibit a U-shaped trajectory in relational matching that is similar to children in the US.

Culturally, Korea shares an emphasis on relationships and situational factors with other East Asian contexts (I. Choi & Nisbett, 1998; I. Choi et al., 2003). But fine-grained analyses caution against treating East Asian societies as homogenous (Zhang et al., 2005). Contemporary Korea has undergone rapid sociocultural change, including globalization and the widespread introduction of early English education (H. Choi, 2023; Lim, 2021; Santos et al., 2017; Shin & Choi, 2008). While Korean adults display a holistic attentional bias overall (Masuda et al., 2008; Masuda & Nisbett, 2001; Miyamoto et al., 2006; Rhode et al., 2016; Tajima & Duffield, 2012), recent work provides evidence that the current generation of Korean parents show increased analytic attentional patterns (Jeong & Choi, 2026; J. Kim & Choi, 2025): when asked to describe various visual scenes, these mothers’ descriptions were biased toward foreground entities, similar to the attentional bias found in North American adults. Critically, the mother’s tendency to focus on objects predicted their preschoolers’ attentional focus on foregrounds (Jeong & Choi, 2026).

Consistent with this shift, Korean caregivers of young infants have adopted more distant parenting strategies (e.g., eye contact, verbal command) than proximal parenting strategies (e.g., bodily contact and guide, direct instruction) relative to parents in collectivistic cultures like Zambia or Turkey (Ross et al., 2017; Yoon & Choi, 2023). Additionally, Korea has undergone a rapid shift in cultural values, especially among younger caregivers, who increasingly value their children’s individual expressions and achievements (Park & Lee, 2004). At the same time, many traditional values remain salient (e.g., respect for elders, social hierarchy) and continue to shape children’s social and epistemic decisions (Beom & Choi, 2020; Oh et al., 2024). These mixed and evolving tendencies could plausibly produce developmental pathways in relational reasoning that differ from both the US and China.

Taken together, this background yields clear, testable alternatives. If lexical bias alone drives the US trajectory of relational reasoning, then Korean learners (who show noun-biased vocabularies beginning around 30 months of age) should more closely resemble their US peers. If broad cultural and/or attentional tendencies are sufficient to sustain relational responding over development, Korean learners should look more like their Chinese peers. If multiple influences interact—for example, verb-rich input in the presence of noun-biased vocabularies, or heterogeneous attentional and parenting practices—then we should observe a unique trajectory in Korea.

### The Present Study

Our study has several related goals. First, we document children’s relational reasoning performance and cognitive bias in a novel context, South Korea, that shares some features of interest with both the US and China. As explained above, this will inform ongoing work investigating which factors may contribute to observed cross-cultural variation in the emergence and developmental trajectory of relational reasoning.

Second, our approach presents an opportunity to evaluate two key predictions of the rational learner account (Walker et al., 2016; Carstensen et al., 2019). Specifically, we should observe (1) early consistency in the emergence of relational reasoning across learning contexts, and (2) later variation in the developmental trajectory, depending on the presence of relevant environmental factors. We evaluate these predictions in three experiments using the cRMTS task. Experiment 1 tests early consistency by measuring whether Korean toddlers (aged 18–30 months) succeed in relational matching, as reported in both the US and China. Experiment 2 measures baseline bias in Korean 3-year-olds using an ambiguous version of cRMTS that pits an object-based solution against a relational one (Carstensen et al., 2019, Exp. 3). Experiment 3 then assesses performance in the standard cRMTS task among Korean 3- and 4-year-olds, and situates these data relative to US children at these ages. To preview our results, our findings bear out predictions about consistency and variation, but with novel and important caveats that we address in the General Discussion.

## 2. Experiment 1: Consistency in Early Emergence

In previous work, toddlers (18–30 months) in the US succeed on a causal version of the Relational Match-to-Sample task (cRMTS), showing genuine conceptual understanding (Walker & Gopnik, 2014, 2017; Goddu et al., 2020), that later declines by around 3 years of age (Walker et al., 2016). Given their early success, the authors argue that children’s ability to infer object-based and relational concepts likely develops in tandem, with the subsequent decline in performance reflecting a failure to *apply* relational reasoning, rather than a lack of competence in inferring *same-different* concepts.

Later, Carstensen and colleagues (2019) found evidence for early success in 18–30-month-olds in China. Unlike their US peers, however, Chinese toddlers show an early advantage for inferring the *same* relation, with the *different* relation emerging somewhat later, between 30 and 36 months of age (see Carstensen et al., 2019, Supporting Information). In Experiment 1, we further examine the claim that relational reasoning is early-emerging by comparing these previous findings with the performance of a novel sample of Korean toddlers.

### 2.1. Method

#### 2.1.1. Participants

A total of 91 Korean-speaking 18–30-month-olds (*M* = 24.0 months; 48 female) in Seoul, South Korea, took part in Experiment 1, with 41 in the *same* condition and 50 in the *different* condition. An additional 17 toddlers were tested but excluded due to failure to complete the task (16) or a technical issue with the stimuli (1). Children were recruited through preschools and public parks, with testing conducted either in the lab or at participants’ homes in and around Seoul. Participants were pseudo-randomly assigned to each condition, and we applied Bayesian optional stopping to determine our sample size (Rouder, 2014), discontinuing data collection when we reached a Bayes factor of 3 in favor of the test (different from chance) or the null hypothesis (chance performance) in a binomial comparison (Kruschke, 2014); we ran this analysis after each day of testing, rather than after each participant, to facilitate data collection outside the lab (recruiting opportunistically at a local museum). If we did not satisfy our criterion, we planned to discontinue data collection at 50 participants per condition, a sample slightly larger than that in Carstensen et al. (2019).

#### 2.1.2. Procedure

The materials and procedure were matched to Walker et al. (2016, Experiment 1) and Carstensen et al. (2019, Experiments 1–2), except that the instructions were presented in Korean (described here in English). The original English instructions were independently translated and back-translated by Korean-English bilinguals, with back-translation checks and adjustments to ensure accuracy.

Children were tested in individual sessions, seated at a table across from the experimenter. After a brief warm-up, the experimenter started the cRMTS task. The experimenter placed pairs of painted wooden blocks on top of a cardboard box, which appeared to play music in response to some pairs and not others. In reality, the experimenter triggered the music by covertly pressing a button to activate a wireless doorbell inside the box. The block pairs exemplified the same relation (matching in shape and color), which activated the box in the *same* condition, or the different relation (mismatched in shape and color), which activated the box in the *different* condition (see Figure 1).

The experimenter began by placing the box on the table, saying, “This is my toy! Sometimes when I put things on top of my toy, my toy plays music, and sometimes when I put things on top of my toy, it does not play music. Let’s see how it works!” The experimenter then introduced a pair of blocks, said “Let’s try!” and placed both blocks on the toy simultaneously. The toy played music, and the experimenter said, “Music!” Then she picked up the blocks and placed them back on the toy, which again played music, saying, “These ones made my toy play music!”

On the second training trial, the experimenter repeated this procedure with a new pair of blocks in the opposite relation (same or different). The new pair did not make the toy play music, and the experimenter responded to the first try with “No music!” and, after the second try, said “These ones did not make my toy play music.” This pattern was repeated with two additional pairs of blocks. The experimenter always began with a causal pair (identical blocks in the *same* condition and blocks of differing colors and shapes in the *different* condition), and alternated inert, causal, inert, using novel blocks in each new pair and randomizing the specific blocks between participants.

After the four training trials, the experimenter said, “Now that you know how my toy works, I need your help finding the things that will make my toy play music! I have two choices for you.” The experimenter then presented the child with two new test pairs of novel blocks, one same pair and one different pair, on either side of the toy. Each pair was displayed on a tray, which the experimenter held up, saying, “I have these… and I have these. Only one of these trays has the things that will make my toy play music. Can you point to the tray that has the things that will make my toy play music?” The trays were placed just out of reach of the child, with the side of the correct pair and order of presentation counterbalanced across participants. The experimenter recorded the child’s first point or reach, and scored the answer as correct if the child chose the test pair (same or different) that corresponded to their training.

### 2.2. Results and Discussion

All data and analysis scripts for these experiments are available at https://osf.io/ue6r3. We used the Bayesian binomial test from our stopping rule to analyze data in each condition. In the *same* condition, 28 of 41 Korean toddlers chose the relationally correct pair of identical blocks (*M* = 68%, *BF*_10_ = 4.07). In the *different* condition, only 25 of 50 toddlers chose the relationally correct pair, performing at chance (*M* = 50%, *BF*_10_ = 0.331). 

Findings from Study 1 indicate that, like their peers in the US and China, toddlers in South Korea successfully infer the relational concept ‘same’ in the cRMTS task. This provides support for cross-cultural generality and aligns with the early consistency prediction of the rational learner account. At the same time, Korean toddlers failed to select the correct relational match in the *different* condition, paralleling the asymmetry observed in Chinese children at the same age (see Figure 5 of this paper and Carstensen et al., 2019, Supporting Information).

Taken at face value, this finding may suggest cultural specificity: perhaps shared features of learning environments in China and Korea advantage the youngest reasoners in the *same* condition relative to the *different* condition. On the other hand, this finding could also reflect cross-cultural consistency—i.e., that reasoning about the ‘same’ relation generally emerges earlier than reasoning about different relations. Indeed, this ‘same’ advantage is observed in US infants (Hochmann et al., 2016, 2018), and similar findings have been reported among 30–36-month-old US children during the initial downward trajectory of the U-shaped curve (Walker et al., 2016). Stated more generally, below-ceiling performance in relational reasoning appears to be associated with an advantage for the same relation in all three cultural contexts. This repeated asymmetry in China, the US, and now in Korea may therefore suggest a universal advantage for the *same* relation.

## 3. Experiment 2: Variation in Relational Bias

In Experiment 1, we find evidence consistent with the first prediction of the rational learner perspective: that young children, who have fewer learned biases, will more consistently engage in relational reasoning across varied contexts. The second prediction of this account, later variation, suggests that performance in older children may diverge to the extent that the context-specific experience that influences the development of their biases differs.^1^ In Experiment 2, we use an ambiguous version of the cRMTS task (Carstensen et al., 2019, Experiment 3) to measure relational or object bias in Korean 3-year-olds that may be relevant for their relational reasoning performance.

### 3.1. Method

#### 3.1.1. Participants

A total of 55 Korean-learning 36–48-month-olds (*M* = 41.2 months; 22 female) and 60 Korean-speaking adults (range: 20–39 years, 38 female) in Seoul and nearby cities in Korea took part in Experiment 2. Participants were again pseudo-randomly assigned to each condition, and we applied Bayesian optional stopping to determine our sample size, discontinuing data collection when we reached a Bayes factor of 3 in favor of the test (different from chance) or null hypothesis (chance performance) in a binomial comparison. If we did not satisfy this criterion, we stopped data collection at 55 participants per condition to match the US sample in Carstensen et al. (2019, Exp 3). One additional child participated but was excluded for failing to follow instructions. Recruitment and testing locations were the same as in Experiment 1.

#### 3.1.2. Procedure

**Figure 2:**
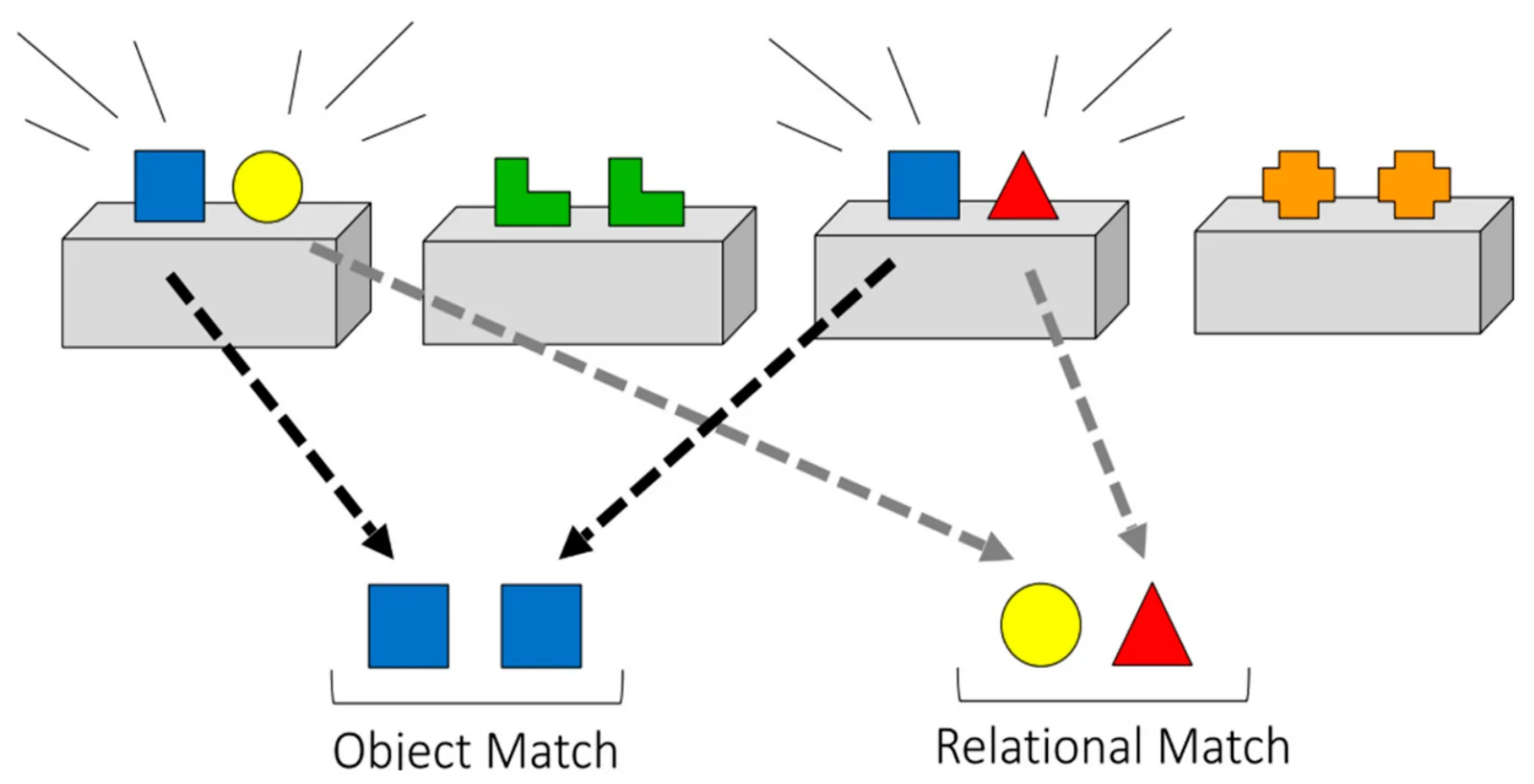
Schematic of the Ambiguous cRMTS training (top row) and test trial (bottom), which pits an object match solution against a relational match. Reproduced from Carstensen et al. (2019). There was not a single correct test choice in this task; participants might infer that a particular block triggered music (e.g., the blue square; in which case, the two blue squares should be the preferred option) or that two blocks in the *different* relation triggered music (in which case, the yellow circle and red triangle should be the preferred option).

Materials were identical to those in Experiment 1. The procedure closely resembled that of the *different* condition, but with three modifications to create an ambiguous causal structure (see Figure 2). First, in the training trials, one of the blocks (represented by the blue square in Figure 2) repeatedly appears in both different pairs that play music. This recurring block provides support for an object-based hypothesis (i.e., the blue square is causal). Second, the test trial was composed of the blocks that were previously observed in the different training pairs, regrouped to create the same (object match) and different (relation match) pairs. Finally, the experiment included only the *different* condition, due to constraints of the study design (i.e., object and relational matches based on the same pair of objects will be identical).

As in Experiment 1, the experimenter asked the child to choose the pair that would make the toy play music. The child’s first point or reach was scored as consistent with either an object selection or a relational selection.

### 3.2. Results and Discussion

**Figure 3:**
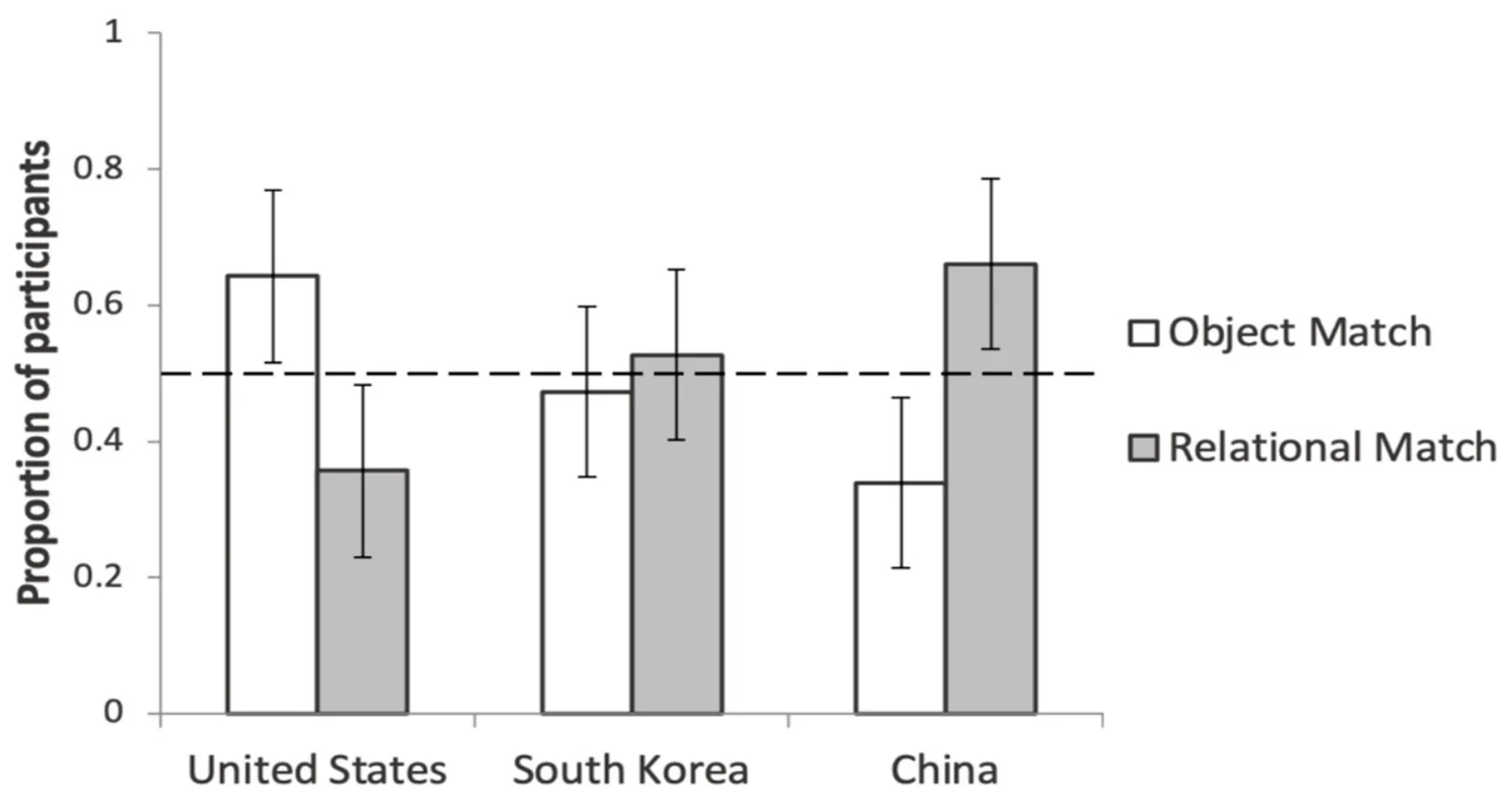
Proportion of object and relational matches selected by South Korean children in the ambiguous matching task (Experiment 2), compared to children from the US and China, as reported in Carstensen et al. (2019). Error bars indicate 95% CIs, and the dotted line indicates chance performance.

As in Experiment 1, we used the Bayesian binomial test from our stopping rule to analyze data in the ambiguous cRMTS task. The Korean 3-year-olds in our sample show intermediate scores in the bias assessment, with 29 of the 55 children choosing the relational match (*M* = 53% relational, *BF*_10_ = 0.34), indicating no clear bias for either objects, as observed in the US, *or* relations, as observed in China (see Figure 3). Notably, Korean adults respond similarly on this task (*n* = 60, *M* = 52% relational, *BF*_10_ = 0.31). This is also consistent with adult performance in the US and China, who show no discernable bias on the ambiguous cRMTS task (Cao et al., 2024).

This result is consistent with the prediction for later variation in relational reasoning, establishing a qualitatively different finding—no observable bias—among 3-year-olds in Korea. This difference in baseline bias across all three countries implies that the factor or factors that are causally relevant for these differences between the US and China further vary in South Korean learning contexts.

## 4. Experiment 3: Variation in Relational Reasoning

Experiment 2 revealed that 3-year-olds in Korea showed no reliable bias toward either relational or object-based solutions in an ambiguous version of the cRMTS task. This finding offers a unique opportunity: it provides a naturally occurring neutral context in which to examine relational reasoning without the influence of strong prior biases. Recall that in previous work, children in the US—who tend to favor object-based solutions—performed poorly on relational reasoning tasks, whereas their counterparts in China—who show a relational bias—performed well (and similarly to younger children in both cultural contexts). Given these opposing patterns, it remains unclear what relational reasoning looks like in the absence of such a bias. Our novel Korean sample therefore provides an opportunity to examine relational reasoning in a population that does not exhibit the clear object or relational bias observed in previous samples.

Importantly, both toddlers and adults typically succeed on the cRMTS task across contexts, despite differing levels of cognitive bias: toddlers perform well before any biases emerge, while adults succeed despite extensive experience that might otherwise privilege object-based reasoning in some cultures. The preschool years, by contrast, represent a period in which relational competence exists, but may be masked by competing cognitive demands or contextual influences (see Walker et al., 2016, Experiments 2–3). Thus, examining the performance of 3-year-olds in Korea allows us to evaluate whether success at this age depends on the presence of a relational bias. If Korean 3-year-olds perform well, success in relational reasoning at this age may reflect a more general developmental capacity. If they fail, it suggests that the cognitive bias observed in Chinese children plays an important role in supporting robust relational reasoning at this age.

Additionally, to further examine the developmental trajectory of relational reasoning, we also extend our sample to include 4-year-olds in both Korea and the United States. This enables us to document performance on cRMTS beyond 48 months for the first time, and to evaluate whether the hypothesized recovery phase of the U-shaped trajectory, previously inferred from adult performance, can be empirically observed across cultural contexts.

### 4.1. Method

#### 4.1.1. Participants

A total of 100 Korean children aged 36–48 months (*M* = 41.5 months; 49 female; 50 per condition), 39 Korean children aged 48–60 months (*M* = 53.4 months; 22 female; 19 in the same condition), and 59 US children aged 48–60 months (*M* = 53.2 months; 28 female; 20 in the *different* condition and 39 in the *same* condition) participated in Experiment 3. Participants were pseudo-randomly assigned to the same or *different* conditions, and we followed the same Bayesian optional stopping protocol as in Experiment 1 to determine the sample size, stopping at a maximum of 50 participants per condition when we did not satisfy our stopping criterion^2^. Recruitment and testing locations within Korea were the same as in the two previous studies. In the US, all participants were recruited and tested at a museum in San Diego.

#### 4.1.2. Procedure

The materials and procedure were identical to those in Experiment 1, with the exception that US children were tested in English.

### 4.2. Results and Discussion

As in the previous studies, we used the Bayesian binomial test from our stopping rule (i.e., a Bayes factor of 3 in favor of the test or null hypothesis) for our analysis. While we do not observe substantial evidence in favor of either hypothesis, our data suggest that performance in Korean 3-year-olds does not differ from chance (*same*, *n* = 50, *M* = 58%, *BF*_10_ = 0.58; *different*, *n* = 50, *M* = 60%, *BF*_10_ = 0.81), and is numerically intermediate to performance in the US and China. By 4 years of age, children in both Korea and the US perform significantly above chance (KR: *same*, *n* = 19, *M* = 79%, *BF*_10_ = 6.18; *different*, *n* = 20, *M* = 85%, *BF*_10_ = 28.11; US: *same*, *n* = 39, *M* = 77%, *BF*_10_ = 60.97; *different*, *n* = 20, *M* = 80%, *BF*_10_ = 8.87).

**Figure 4:**
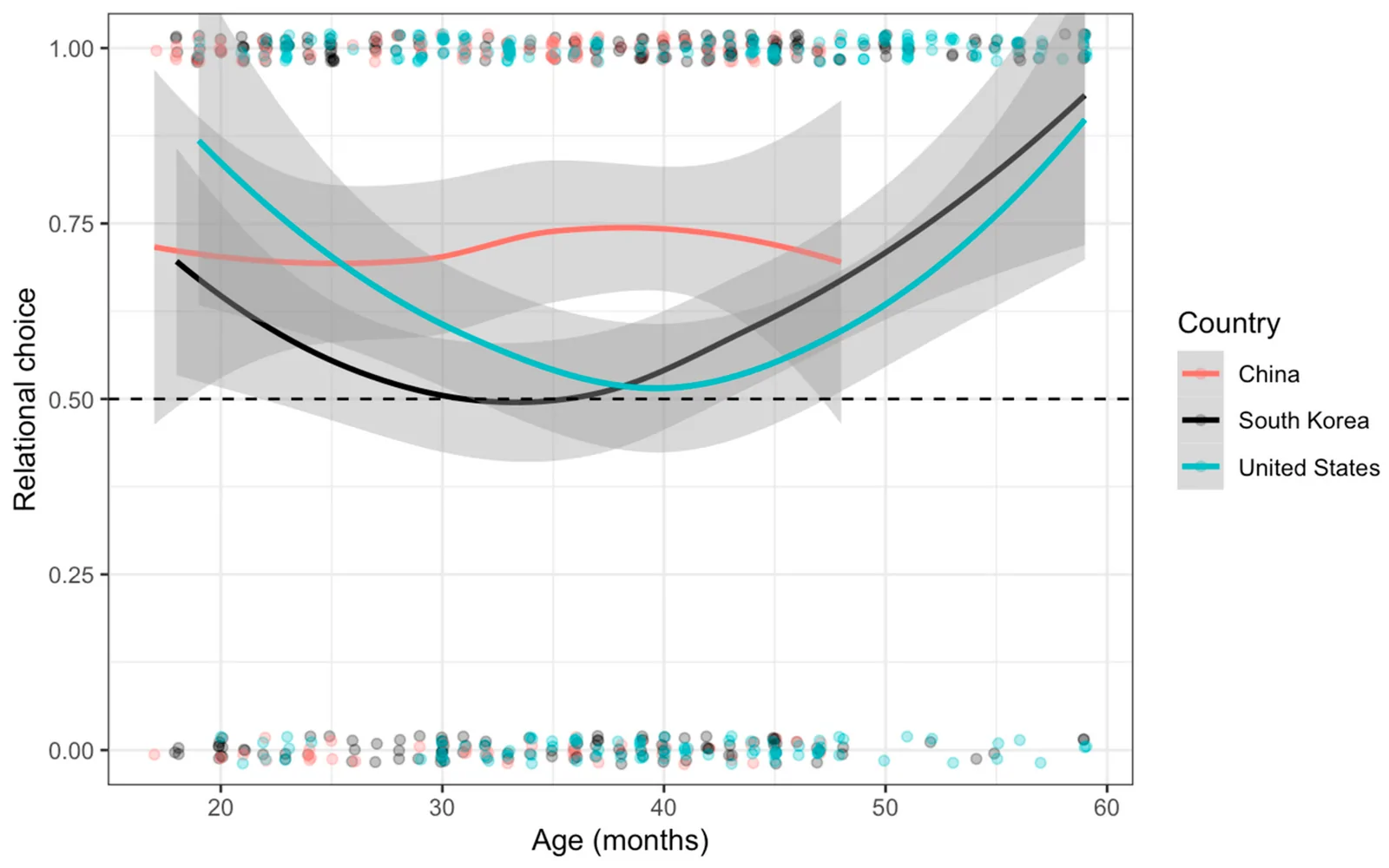
Overall relational matching trajectories, including data for 18–47-month-olds in the US and China reported in Carstensen et al. (2019), plotted with Loess-smoothed fit lines. Shaded regions indicate 95% CIs.

We also compared our cross-sectional data in this paradigm (Experiments 1 and 3) from children between 18 and 47 months with comparable data from children in China and the US reported in Carstensen et al. (2019); see Figure 4. For this, we fit a Bayesian logistic regression predicting cRMTS performance as a function of age (in months, centered), quadratic age (to account for U-shaped trajectories^3^), condition (same or different), country (Korea, US, or China), and their interactions.

**Figure 5:**
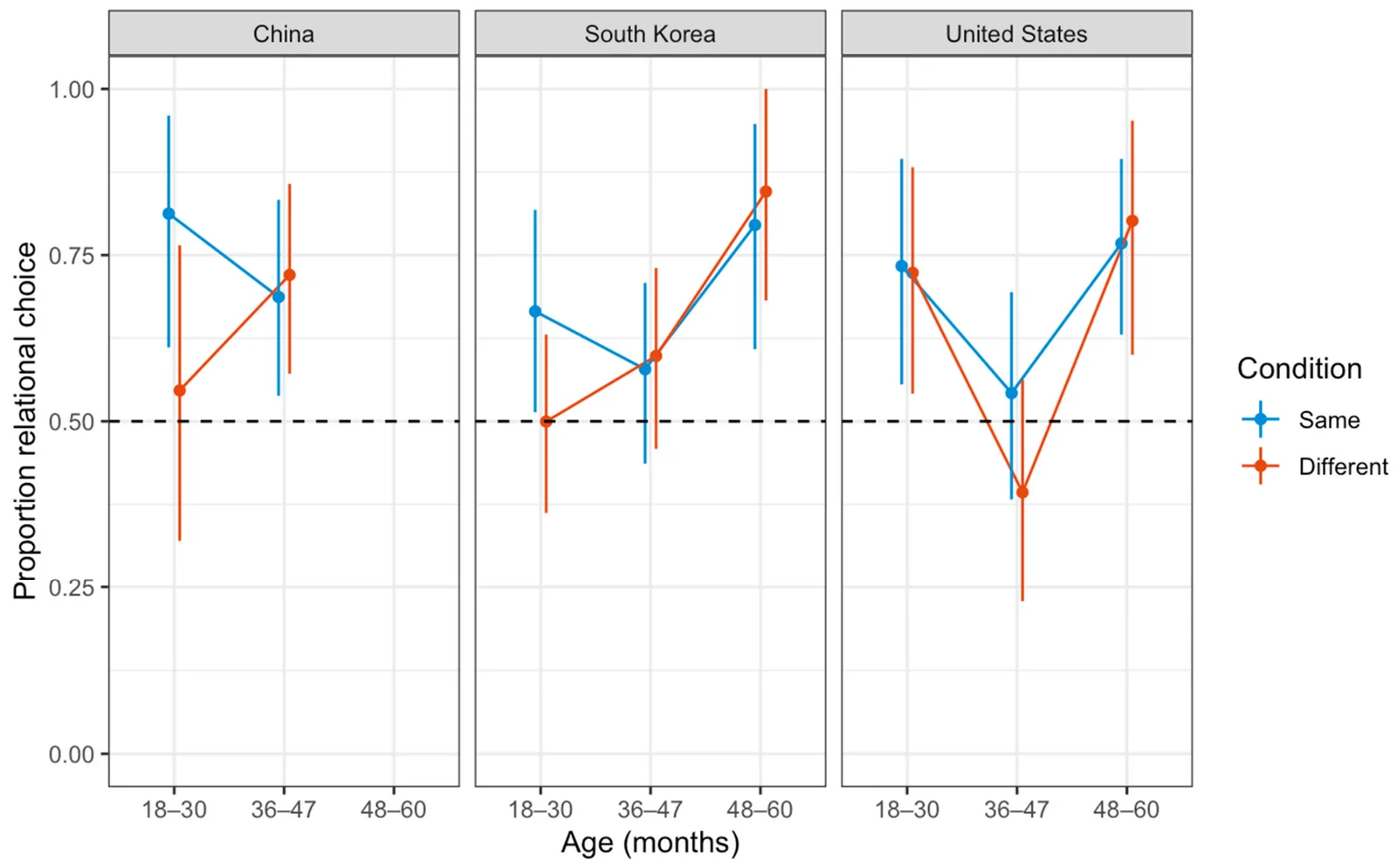
Mean performance in each country plotted according to the age and condition bins in our main analysis. Error bars show 95% CIs.

There was a main effect of the quadratic age term (*β* = −0.12, 95% Credible Interval [−0.23, −0.03]^4^), and significant 2-way interactions between quadratic age and condition (*β* = 0.15 [0.03, 0.28], with different as reference condition); age and country for both Korea (β = 0.37 [0.00, 0.77]) and China (β = 0.80 [0.13, 1.65]) compared to the US; and also quadratic age and country for Korea (β = 0.13 [0.01, 0.26]) and China (*β* = 0.26 [0.09, 0.46]). There were significant 3-way interactions between age, condition, and country for both Korea (*β* = −0.77 [−1.31, −0.25]) and China (*β* = −1.15 [−2.06, −0.38]), compared to the US. Finally, there were also significant 3-way interactions between quadratic age, condition, and country for both Korea (β = −0.21 [−0.37, −0.06]) and China (*β* = −0.33 [−0.55, −0.14]), compared to the US (see Figure 5).

Our binomial analysis does not support the null or test hypotheses for 3-year-olds in Korea, though we find clear evidence that 4-year-olds in Korea and the US succeed in choosing the relationally correct solution in both the *same* and *different* conditions. Figure 4 shows overall performance at all ages for children in our Korean sample, alongside comparable data from their peers in the US and China, combined across conditions. This is the first empirical documentation of the full trajectory of the U-shaped curve in the US, which had been previously hypothesized on the basis of adult performance in this task and success in related reasoning tasks by preschool and school-aged children. Within and across conditions, the developmental trajectory observed in our Korean sample qualitatively resembles that in the US. And indeed, the overall performance in both countries is better characterized by models that incorporate a quadratic age term in addition to a linear one, enabling U-shaped model fits of the type shown in Figure 4.

## 5. General Discussion

The present research examined the early development of relational reasoning among Korean children aged 18–60 months, providing a new test of the rational learner framework across a distinct cultural and linguistic context. Consistent with this account’s core predictions, we find evidence for early consistency—18–30-month-olds in Korea, like their peers in the US and China, show early success in relational reasoning—and later variation, as cognitive bias diverges over the preschool years. We also measure the performance of 4-year-olds in the US and Korea, who show robust success, providing evidence for the upswing in a hypothesized U-shaped learning trajectory characterizing US performance. Further, by comparing Korean children to previously studied samples from the US and China, we offer new evidence that the developmental trajectory of relational reasoning is neither universal nor binary, but instead likely reflects the interaction of multiple environmental influences that shape how relational understanding is deployed.

At the broadest level, Korean children’s performance shows similarities to that of children in the US and China, both in terms of early relational matching and later relational bias. This pattern aligns with the prediction that when relevant environmental inputs differ, so too will the priors that guide children’s inferences. According to the rational learner account (Walker et al., 2016; Carstensen et al., 2019), children integrate information from the learning environment with prior expectations to infer which type of evidence is most likely to be informative. Our data suggest that Korean children may occupy a developmental and cultural “middle-ground”: their early success on cRMTS and later neutrality in cognitive bias imply that their priors may be influenced by both object-focused and relation-focused experience, yielding a unique developmental trajectory.

The lack of bias we observe among Korean 3-year-olds in Experiment 2 is a particularly serendipitous finding, as it presents something of a natural control condition to compare with learning contexts like the US and China. In prior work, strong performance in relational reasoning has been confounded by clear differences in cognitive bias toward relational or object-based solutions: US children show a bias toward objects and perform poorly in the relational task, while Chinese children show a bias for relations and perform well. It therefore remained unclear whether a relational bias is necessary to support robust relational reasoning in preschool-aged children. Here, in the absence of a relational bias, Korean children’s performance resembles that of US children, in that they do not reliably choose relational solutions on the cRMTS task. This finding suggests that the bias observed in Chinese children may play a causal role in supporting robust relational reasoning at this age.

That said, the two-answer forced-choice design of the ambiguous cRMTS task limits our ability to interpret children’s responses in Experiment 2 as definitive evidence for a “neutral” stance in Korean 3-year-olds. It is possible that this pattern of responding reflects their failure to understand the task, rather than a true lack of bias. Given Korean toddlers’ success in Experiment 1, the interpretable data collected from 3-year-olds in the US and China on the same ambiguous task, and success in 4-year-olds in both the US and Korea, we believe this alternative interpretation is unlikely. While additional work is necessary to discriminate these possibilities, we note that both possibilities—a neutral bias or random responding—present cross-cultural diversity at this age, given the clear object and relational biases demonstrated by children in the US and China, respectively.

Although our findings cannot isolate the specific type of input that is responsible for the intermediate pattern observed in Korean children (or for shaping the broader differences in the trajectory of performance across cultural contexts), these data are consistent with existing claims linking the dip in relational reasoning to noun learning (e.g., Hoyos et al., 2016). While labeling objects’ names or relations has been shown to disrupt or support relational reasoning among US 3-year-olds (Christie & Gentner, 2014), similar labeling interventions did not affect the abstraction of *same–different* relations among 12-month-old infants (Anderson et al., 2022), suggesting that language learning may have a greater impact on reasoning during the preschool years. In line with this possibility, a recent study reported that Korean 3-year-olds’ relational abstraction on the cRMTS task was positively linked with their grammar score on the MacArthur-Bates Communicative Inventory (J. Kim & Choi, 2025). In contrast, relational reasoning was negatively associated with their vocabulary score. This provides additional support for dynamic influences from language learning; it is consistent with the possibility that noun learning hinders relational abstraction and structural learning scaffolds performance.

On the other hand, a wide range of other linguistic and cultural factors are implicated in relational reasoning, many of which interact over development. For instance, while vocabulary data reveal a noun-biased pattern of word learning in Korean that parallels English (Frank et al., 2021), input from Korean caregivers more closely resembles that of Chinese caregivers in its relative emphasis on verbs and actions (Hung & Chang, 2023; M. Kim et al., 2026). This apparent mismatch between language input and word learning suggests that more detailed measures and nuanced explanations may be needed to account for developmental differences in language learning. These empirical complexities also appear in work documenting attentional and interpretive tendencies across cultures. Korea’s rapid sociocultural change—marked by increasing Westernization (Santos et al., 2017), more pronounced individualism in family structures and language use (H. Choi, 2023), early individual achievement-focused education (Park & Lee, 2004), and hybrid caregiver visual attention (Jeong & Choi, 2026; J. Kim & Choi, 2025) and child-rearing practices (Yoon & Choi, 2023)—could produce mixed biases that jointly influence children’s relational reasoning.

It is also possible that the intermediate performance we observe in the Korean sample reflects a difference in developmental timing, rather than a qualitatively distinct trajectory. Perhaps Korean children also develop an object bias and follow the U-shaped curve observed in the US, but progress through these phases at a different pace due to variation in the onset of this bias. While our cross-sectional data cannot definitively distinguish between explanations appealing to the timing versus the type (or shape) of the trajectory, our inclusion of 3- and 4-year-olds allows us to begin empirically tracing this curve. The success of 4-year-olds in both Korea and the U.S. provides the first direct evidence of the upswing in relational reasoning that had previously been hypothesized on the basis of adult performance alone.

Importantly, the work reported here is correlational, confounding potential effects of language and culture with sample characteristics. It is also empirically sparse in comparison to complete accounts of relational reasoning, which likely emerges from interacting cognitive systems including executive function, working memory, attention, categorization, processing efficiency, language, and metacognitive regulation. As noted in the introduction, prior work suggests that preschoolers’ relational reasoning depends jointly on relational language use and working memory (Esmer et al., 2025), executive function (Richland et al., 2010), and broader cognitive functioning, which may also vary over development (Demetriou et al., 2025, 2026). These various findings highlight a limitation of treating relational reasoning as a unitary capacity without also measuring its component cognitive processes. Further, these processes may contribute differently over development. For example, improvements in executive control may help children inhibit salient object features, while improvements in working memory may support maintaining multiple relational representations simultaneously. Likewise, language may influence the availability of relational representations themselves. Because the present design measures only behavioral performance, it cannot distinguish among these possibilities.

To develop a more comprehensive account of relational reasoning across development and cultures, future research should assess these component processes in cross-sectional^5^ and, ideally, longitudinal designs to identify which cultural factors influence the cognitive mechanisms that, in turn, support relational reasoning. This would likely require a coordinated effort to establish cross-culturally validated developmental measures of these processes, but may become tractable through large-scale collaborative infrastructure projects (Frank et al., 2025; Kachergis et al., 2025). It is our hope that the initial cross-cultural findings reported here, together with our review of potential explanations for these differences, illustrate the value of such comprehensive approaches.

Taken together, our findings refine and extend the rational learner account by evaluating its predictions in a new cultural context. We refine prior predictions of early consistency and contribute to an emerging set of findings (Goddu et al., 2020; Hochmann et al., 2016, 2018; Walker et al., 2016; Lapidow et al., 2025) that suggest greater facility for reasoning about the *same* relation compared to the *different* relation. We also extend the rational learner account by documenting additional variation in older children’s relational reasoning. In measuring performance across development in a third cultural context, we observe a third pathway. This preliminary evidence suggests that, rather than a single universal developmental trajectory or a bifurcation between object-biased and relational pathways, variation may be the most prominent pattern in the development of relational reasoning across cultures. These findings highlight the importance of studying development in diverse cultural contexts to understand how universal cognitive capacities like abstract thinking are tuned by experience to fit the structure of early learning environments.

## Data Availability

The data presented in this study are openly available in [OSF] [https://osf.io/ue6r3].

## Notes

1 NB: the present study is not designed to evaluate contextually varying factors that may cause differences in relational reasoning, or to measure contextual variation between the U.S., South Korea, and China—these are remaining empirical questions—our goal here is to document bias in relational reasoning in a novel population to inform our general understanding of variation in relational bias, given that little data has been collected with children outside of English-speaking contexts in the U.S.

2 There were two exceptions to this policy in US recruitment: (1) we accidentally overrecruited US children in the same condition because of a miscommunication about the stopping rule, running 39 when we would have stopped at 23 if we had followed our plan, and (2) we intentionally overincluded US children in the different condition because we met our stopping rule at 10 participants, but followed reviewer feedback to include a minimum of 20 participants per condition; in both cases, our results are qualitatively similar for the smaller and larger samples and we therefore report statistics for the larger samples. We also implemented this 20-participant minimum with our Korean samples after meeting our stopping rule with 8 4-year-olds in each condition, overrecruiting to meet this minimum of 20 in the different condition, with 19 in the same condition because of a duplicate entry; again, our results are qualitatively similar for the smaller and larger samples and we therefore report statistics for the larger samples.

3 We also fit a version of this model without the quadratic age term, but the model with quadratic age outperformed the simpler alternative in model comparison (*χ*^2^(6) = 15.86, *p* = 0.015).

4 The credible interval indicates the range within which 95% of posterior draws fall. As a practical rule of thumb, an effect whose 95% credible interval excludes zero can be interpreted similarly to a statistically significant result (roughly analogous to *p* < 0.05), though this is an informal heuristic rather than a strict equivalence.

5 See Carstensen et al. (in press) and Boccalato-Rodríguez et al. (2026) for cross-sectional cross-cultural studies of multiple factors including attention, social cognition, and relational reasoning.
